# Screening for phenotype selective activity in multidrug resistant cells identifies a novel tubulin active agent insensitive to common forms of cancer drug resistance

**DOI:** 10.1186/1471-2407-13-374

**Published:** 2013-08-06

**Authors:** Mårten Fryknäs, Joachim Gullbo, Xin Wang, Linda Rickardson, Malin Jarvius, Malin Wickström, Saadia Hassan, Claes Andersson, Mats Gustafsson, Gunnar Westman, Peter Nygren, Stig Linder, Rolf Larsson

**Affiliations:** 1 Department of Medical Sciences, Division of Clinical Pharmacology, Uppsala University, S-751 85 Uppsala, Sweden; 2 Department of Oncology, Radiology and Clinical Immunology, Uppsala University, S-751 85 Uppsala, Sweden; 3 Department of Oncology-Pathology, Cancer Center Karolinska, Karolinska Institute, Stockholm, Sweden; 4 Department of Chemical and Biological Engineering, Chalmers University of Technology, S-412 96 Gothenburg, Sweden

**Keywords:** Screening, Myeloma cell lines, Primary cultures, Drug resistance, Tubulin inhibition

## Abstract

**Background:**

Drug resistance is a common cause of treatment failure in cancer patients and encompasses a multitude of different mechanisms. The aim of the present study was to identify drugs effective on multidrug resistant cells.

**Methods:**

The RPMI 8226 myeloma cell line and its multidrug resistant subline 8226/Dox40 was screened for cytotoxicity in response to 3,000 chemically diverse compounds using a fluorometric cytotoxicity assay (FMCA). Follow-up profiling was subsequently performed using various cellular and biochemical assays.

**Results:**

One compound, designated VLX40, demonstrated a higher activity against 8226/Dox40 cells compared to its parental counterpart. VLX40 induced delayed cell death with apoptotic features. Mechanistic exploration was performed using gene expression analysis of drug exposed tumor cells to generate a drug-specific signature. Strong connections to tubulin inhibitors and microtubule cytoskeleton were retrieved. The mechanistic hypothesis of VLX40 acting as a tubulin inhibitor was confirmed by direct measurements of interaction with tubulin polymerization using a biochemical assay and supported by demonstration of G2/M cell cycle arrest. When tested against a broad panel of primary cultures of patient tumor cells (PCPTC) representing different forms of leukemia and solid tumors, VLX40 displayed high activity against both myeloid and lymphoid leukemias in contrast to the reference compound vincristine to which myeloid blast cells are often insensitive. Significant *in vivo* activity was confirmed in myeloid U-937 cells implanted subcutaneously in mice using the hollow fiber model.

**Conclusions:**

The results indicate that VLX40 may be a useful prototype for development of novel tubulin active agents that are insensitive to common mechanisms of cancer drug resistance.

## Background

Current treatment strategies for treatment of cancer are limited by the occurrence of drug resistance [1-3]. The cellular mechanisms have been extensively studied in cell line models and include alterations of drug transport, metabolism, DNA synthesis and repair, cell survival and apoptosis. Both genetic and epigenetic changes may be involved in determining the balance between drug sensitivity and resistance [4,5]. Consequently, novel therapies avoiding these mechanisms are urgently needed.

During the past decades most screening approaches for identification of new cancer drug candidates have utilized cell free assays for detection of specific interactions with known or emerging molecular targets [6]. However, the relatively poor outcome with respect to identification of clinically novel and significantly improved cancer drugs has led to a renewed and growing interest for cancer drug screening based on compound induced changes in cellular phenotypes [7-9]. Cultures of human tumor cell lines have been the general model in these efforts and are important tools for predicting mechanisms of drug action as demonstrated in numerous reports [7,9]. Furthermore, recent results utilizing very large panels of cell lines indicate that they also to a large extent retain genomic features of the primary tumor and can recapitulate clinical findings with regard to their response to targeted inhibitors [9].

We have previously utilized the myeloma cell line RPMI 8226 and its multidrug resistant (MDR) 8226/Dox40 subline for phenotype selective activity in response to an annotated compound library [10]. The 8226/Dox40 subline over expresses P-glycoprotein [11], but also other mechanisms are likely contributing to the multidrug resistant phenotype [12]. We have also previously demonstrated that over expression of STAT1-regulated genes contribute to doxorubicin resistance observed in 8226/Dox40 cells [13,14].

In the present study the same myeloma cell lines were tested in response to 3,000 chemically diverse compounds to explore the possibility of finding compounds selectively active against the MDR phenotype. After hit validation and counter screening one hit compound, VLX40, was selected for mechanistic investigation and further preclinical evaluation.

## Methods

### Cell culture

For primary screening RPMI 8226 and its multidrug resistant cell line 8226/Dox40 were used. In a secondary screen, a cell line panel representing different drug resistance phenotypes was used (described in Table 1). The cell lines of this panel were cultured and harvested as previously described [14].

**Table 1:** Cell line panel representing different types of drug resistance

**Cell line**	**Diagnosis**	**Selective agent**	**Mechanism of resistance**	P-gp expression*	**Reference**
RPMI 8226	Myeloma			82	
8226/Dox40	Myeloma	Doxorubicin	P-gp170	27002	[11,15]
8226/LR5	Myeloma	Melphalan	GSH associated	Not expressed**	[16]
CCRF-CEM	T-cell leukemia			Not expressed**	
CEM/VM-1	T-cell leukemia	Teniposode	Topo II associated	Not expressed**	[17]
NCI-H69	Small cell lung cancer			Not expressed**	
H69AR	Small cell lung cancer	Doxorubicin	MRP associated	Not expressed**	[18]
U-937	Histiocytic lymphoma			Not expressed**	
U-937-vcr	Histiocytic lymphoma	Vincristine	Tubulin associated	875	[19]

*mRNA gene expression, probe ID 209993_at (ABCB1 P-gp), (Affymetrix, Inc.).

**Gene expression not detected, absent call according to MAS5 (Affymetrix, Inc.).

An additional 98 primary cultures of primary human tumor cells (PCPTCs) from different tumor types, and four preparations of normal peripheral blood mononuclear cells (PBMC), detailed in Table 2, were used to determine the activity spectrum of VLX40 and, for comparison, six standard cytotoxic drugs chosen to represent different mechanistic classes. The tumor samples were obtained by bone marrow/peripheral blood sampling, routine surgery or diagnostic biopsy. Leukemic cells and PBMCs were isolated by 1.077 g/ml Ficoll-Paque centrifugation [20]. Tumor tissue from solid tumor samples was minced into small pieces and tumor cells were isolated by collagenase dispersion followed by Percoll density gradient centrifugation [21]. The patient sampling was approved by the Regional Ethics Board, Uppsala, Sweden. Cell viability was determined by trypan blue exclusion test and the proportion of tumor cells in the preparation was judged by inspection of May-Grunwald-Giemsa stained cytospin slides. All samples used in this study contained more than 70% tumor cells.

**Table 2:** **Median IC**_**50 **_**for different diagnoses in response to VLX40**

**Diagnosis**	**Median IC**_**50 **_**(μM)**	**Number of samples**
Breast cancer	>34	8
Colon cancer	>34	5
Lung cancer	>34	6
Renal cancer	>34	8
Ovarian cancer	>34	19
AML	1.56	8
ALL	3.21	19
CML	0.98	2
CLL	2.18	11
NHL	0.51	13
PBMC	16.3	4

The human cell lines used for mechanistic studies were MCF7 (breast cancer), HCT 116 (colon cancer) and hTERT-RPE-1 (normal epithelial cell line). MCF7, HCT 116 and HL-60 were obtained from American Type Culture Collection (ATCC, Rockville, MD) whereas hTERT-RPE-1 was from Clontech (Palo Alto, CA). In the in vivo hollow fiber studies the myelocytic cell line U-937 was used. The normal epithelial hTERT-RPE-1 cells were cultured in Dulbecco’s Modified Eagles Medium nutrient mixture F-12 Ham, supplemented with 10% heat-inactivated fetal calf serum, 2 mM glutamine, 100 μg/ml streptomycin and 100 U/ml penicillin (all from Sigma Aldrich Co, St Louis, MO) at 37°C in humidified air containing 5% CO_2_. MCF-7 was grown in in Eagle’s Minimal Essential Medium, supplemented as above. HCT116 were grown in complete McCoy’s medium. RPMI 8226, 8226/Dox40, HL-60 and U-937 were grown in complete RPMI medium.

### Preparation of compounds for screening

The Maybridge Hitskit 3000 library (Maybridge Inc) consists of 3000 chemically diverse compounds. The library was delivered in 36 racks each containing 80 compounds dissolved in DMSO to 10 mg/ml. For the screening, aliquots of the DMSO solutions were transferred to 96-well plates and were further diluted with PBS to obtain stock solutions of 100 μg/ml from which four different 384-well plates for screening were prepared with final test concentrations of 1 μg/ml. In all steps, the Biomek 2000 pipetting station connected to a plate stacker carousel (Beckman Coulter Inc, Fullerton, CA) in a safety cabinet (Bigneat Inc, Hampshire, UK) was used. For dose-response studies, plates containing VLX40 (Vivolux AB, Uppsala, Sweden) and other compounds were prepared by 10-fold serial dilutions in the concentrations 0.004 to 40 μM using the same robotic system. The plates were stored at -70°C until further use. The screening identified one compound with higher activity against 8226/Dox40 cells compared to its parental counterpart RPMI 8226. This compound, chemically a quinoline alkaloid (2-phenyl-4-hydroxyquinoline-6-carboxylic acid ethyl ester), was designated VLX40, and subjected for detailed studies.

### Measurement of cancer drug activity

The Fluorometric Microculture Cytotoxicity Assay, FMCA, described in detail previously [22], was used for measurement of the cytotoxic effect of library compounds and the established standard drugs. The FMCA is based on measurement of fluorescence generated from hydrolysis of fluorescein diacetate (FDA) to fluorescein by cells with intact plasma membranes. Cells were seeded in the drug-prepared 384-well plates using the pipetting robot Precision 2000 (Bio-Tek Instruments Inc., Winooski, VT). The number of cells per well was 2,500 - 5,000 for solid tumor samples and 10,000 – 20,000 for leukemic samples. In each plate, two columns without drugs served as controls and one column with medium only served as blank.

The plates were incubated for 72 h and then transferred to an integrated HTS SAGIAN Core System consisting of an ORCA robot (Beckman Coulter) with CO_2_ incubator (Cytomat 2C, Kendro, Sollentuna, Sweden), dispenser module (Multidrop 384, Titertek, Huntsville, AL), washer module (ELx 405, Bio-Tek Instruments Inc), de-lidding station, plate hotels, barcode reader (Beckman Coulter), liquid handler (Biomek 2000, Beckman Coulter) and a multipurpose reader (FLUOstar Optima, BMG Labtech GmbH, Offenburg, Germany) for automated FMCA. Quality criteria for a successful assay included a mean coefficient of variation of less than 30% in the control wells and a fluorescence signal in control wells of more than 5 times the blank (10 times for cell lines). Survival index (SI) is defined as the fluorescence of test wells in percentage of controls with blank values subtracted.

### Multiparametric high content evaluation of apoptosis and cell cycle arrest

The fluorescence microscope ArrayScan High Content Screening (HCS) system (Cellomics Inc., Pittsburgh, PA, USA) was used to study apoptosis and cell cycle arrest. For these assays, cells were seeded into 96-well plates (PerkinElmer Inc., Wellesley, MA, USA), left to attach over night, before test compounds were added.

Cell death characteristics were studied using a multi-parametric HCS assay described in detail previously [23]. Apoptosis was evaluated after 6, 24 and 48 h exposure to VLX40 in MCF-7 cells. The FLICA probe FAM-DEVD-FMK (carboxyfluorescein-labeled fluoromethyl ketone peptide inhibitor of caspase-3; at a final concentration of 20 μM) was added 1 h before the end of the drug exposure to stain activated caspase-3/7. Plates were then washed and nuclei stained with 10 μM Hoechst 33342 in a fixation solution with 3.7% formaldehyde.

To study cell cycle arrest, HCT116 cells were incubated for 24 h with VLX40. Cells were stained using Cell Cycle Kit I reagents for DNA content and phospho-histone H3 staining (Thermo Fisher Scientific) according to the manufacturer’s instructions. Primary antibodies specific for phospho-histone H3 (rabbit), secondary antibodies DyLight 549 Conjugated Goat anti-Rabbit IgG and DAPI dye were used.

Processed plates were loaded in the ArrayScan and analyzed. Images were acquired for each fluorescence channel, using suitable filters with 10X or 20X objective and in each well at least 1000 cells were analyzed. Quantification of apoptosis was performed by measuring caspase-3 activation and nuclear fragmentation, wheras quantification of cell cycle arrest was obtained by nuclear DNA content (mean average intensity of DAPI) and phospho-histone H3 (total intensity).

### Flow cytometry analysis of cell cycle and apoptosis

Cells were seeded in 24- well plates 24 h prior to treatment with different concentrations of VLX40 for 6, 16, 24 and 48 hours. Upon drug exposure, cells were washed with PBS and stained with Annexin V-FITC according to the instructions of the vendor (Annexin V-FITC apoptosis detection kit 556547, BD Pharmingen). Cell cycle analysis was performed by labeling digitonin-permeabilized cells with 5 ug/ml propidium iodide. Flow cytometry analysis was performed using a BD LSR II flow cytometer.

### Phase contrast microscopy

Time-lapse phase contrast microscopy was performed using an automated IncuCyte phase contrast microscope (Essen Instruments, Ann Arbor, MI). MCF-7 cells (10,000/well) were plated on 24-well ImageLock plates (Essen Instruments) and immediately placed into the IncuCyte imaging system. The chamber is designed to fit into a standard, humidified incubator in an atmosphere of 5% CO_2_, and a moving objective allows the cell culture to be stationary while images are captured at different positions from well to well. Images were collected at 1 h intervals starting 30 min after placing the plate in the IncuCyte chamber and cells were left to attach for 24 h when drug treatment was performed. Cell density (i.e. confluence) was calculated using the IncuCyte software.

### Microarray analysis

RNA from cell cultures was isolated using RNeasy Mini Kit from Qiagen and immediately stored at -70°C until further use. RNA purity and quality was measured using an ND 1000 spectrophotometer (NanoDrop Tecnhologies, Wilmington, DE) and Bioanalyzer 2100 (Agilent Technologies Inc, Palo Alto, CA, USA), respectively. Starting from 2 μg of total RNA, gene expression analysis was performed using Genome U133 Plus 2.0 Arrays according to the GeneChip Expression Analysis Technical Manual (Rev. 5, Affymetrix Inc., Santa Clara, CA). Raw data was normalized using MAS5 (Affymetrix Inc.). Connectivity Map (cmap) build 02 (http://www.broad.mit.edu/cmap) contains genome-wide expression data for 1,309 compounds (6,100 entries, including replicates, different doses and cell lines). The original protocol using MCF-7 breast cancer cells as described by Lamb et al. was used [24]. Briefly, cells were seeded in a 6-well plate at a density of 0.4 × 10^6^ cells per well. Cells were left to attach for 24 h, followed by exposure to either VLX40 at a final concentration of 10 μM, or to vehicle control (DMSO). After 6 h the cells were washed with PBS and total RNA was prepared. Gene expression ratios for drug treated vs. control cells were calculated to generate a list of regulated genes. This list was further filtrated using the flags from the MAS5 normalization. Only probes with signals over 300 arbitrary units and present call in both VLX40 treated and vehicle control were used in the Gene Set Enrichment Analysis (GSEA). In the cmap analysis, only probes present on HG U133A were used, for cmap compatibility. The 20 most up and the 10 most down regulated genes (i.e. probes) were uploaded into the cmap and compared to the 6,100 instances in the cmap database, to retrieve a list of compounds with similar response profile as VLX40. The GSEA software and method for microarray result exploration has been described elsewhere [25]. Briefly, the pre-ranked list (VLX40 exposed MCF-7 cells vs. untreated control, described above) was compared to *a priori* defined and curated gene sets (C2). The p-value refers to the nominal p-value after 1000 permutations.

### Measurements of tubulin polymerization

Tubulin polymerization from purified tubulin monomers was measured as increased fluorescence because of the incorporation of a fluorescent reporter into growing microtubules. All reagents necessary for performing the assay were provided in the kit BK011 from Cytoskeleton (Denver, Colorado, USA). The fluorescence was measured at 1-min intervals for 60 min using a FLUOstar Optima (BMG Labtech GmbH, Offenburg, Germany).

### Immunological assays

Spheroids produced by the hanging drop method in 96 well plates were fixed in paraformaldehyde, dehydrated, embedded in paraffin and sectioned and stained for Ki67 and active caspase-3, as previously described [26].

### In vivo studies

Myeloid U-937 cells were cultured inside semi-permeable polyvinylidene fluoride fibers and assessed in the hollow fiber assay [27,28]. The fibers were implanted subcutaneously into the back of immunocompetent animals (male NMRI mice, Scanbur, Sollentuna Sweden). The following day each mouse was treated with a single subcutaneous injection of VLX40 at a dose of either 0.5 μmol/animal (n = 8), 2 μmol/animal (n = 8), or vehicle (n = 8). Fibers were retrieved after 6 days and cell density evaluated using the MTT (3-[4,5-dimethylthiazol-2-yl]-2,5-diphenyltetrazolium bromide)-assay [29]. The method is based on the conversion of MTT to blue formazan crystals by living cells. The formazan was extracted by DMSO as previously described [28], and optical density (OD) read at 570 nm. Cell density for each fiber on retrieval day was expressed as net growth, defined as (OD retrieval day – OD implantation day)/OD implantation day × 100, i.e. the percent change in cell density in the fibers during the 6 days of *in vivo* experiment. The animals were observed regarding behavior and weight gain throughout the experiment. 200 μl blood samples were obtained through the orbital plexus after anesthetization with isofluran just before euthanasia, and analyzed for hematological parameters. Animals were caged four in each cage and fed a commercial diet (Lactamin AB, Sweden), with water given ad libitum. The study was approved by the Animal Ethics Committee in Uppsala, Sweden.

### Data analysis and statistics

Screening data was exported to Vortex (Dotmatics Inc, UK) software for analysis. A Survival Index of less than 50% in myeloma 8226/Dox40 and more than 50% in parental RPMI 8226 cells was set as the criteria for qualifying as a hit compound.

Concentration-response data of screening hits and standard agents were analyzed using the software GraphPadPrism4 (GraphPad Software Inc., San Diego, CA, USA). Data was processed using non-linear regression to a standard sigmoidal dose-response model to obtain IC_50_-values (the concentration resulting in a SI of 50%).

Response rate in PCPTCs of a specific diagnosis was defined as the fraction of samples having an SI below the median, calculated from all PCPTSs included in the study, at the drug concentration showing the largest SD in survival (SI). For VLX40 this concentration was 3.4 μM. The data for the reference compound vincristine was taken from Lindhagen et al [30], and recalculated as response rate at 1 μM. The PCPTC samples used are listed in Table 2. The relative effect of a drug on solid compared with hematological tumors was indicated by the S/H ratio, defined as the ratio between the total response rates for the solid and the hematological samples. Tumor cell specific activity was estimated by calculation of the ratio of the median IC_50_-value for PBMC over that of chronic lymphocytic leukemia (CLL) samples. Comparisons between groups in the hollow fiber experiment were done with Student’s *t*-test.

## Results

### Drug screening using multidrug-resistant myeloma cells

**Figure 1:**
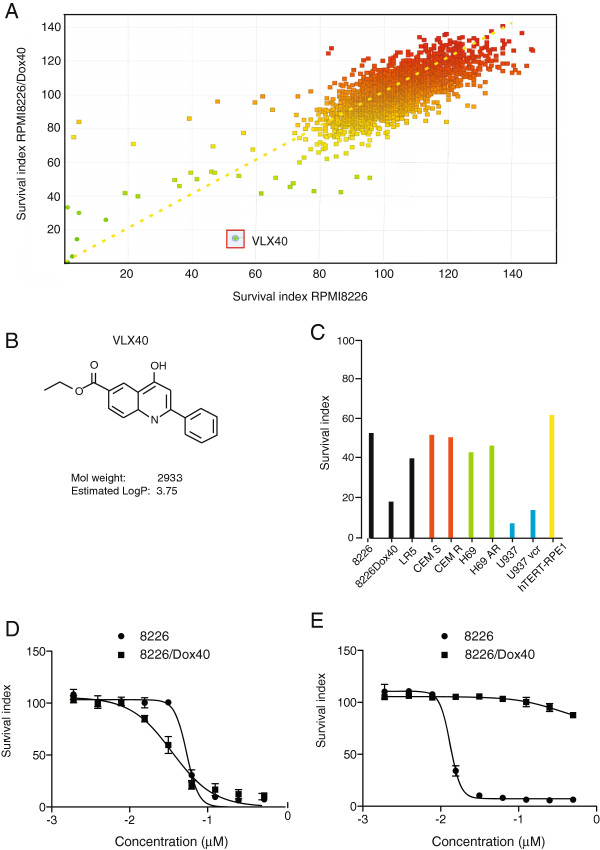
**Drug screening in myeloma cell lines. (A)** The overall screening results are displayed and expressed as survival index with results for 8226/Dox40 displayed on the Y axis and the parental RPMI 8226 cells on the X-axis. **(B)** Molecular structure and chemical properties of VLX40 **(C)** Activity of VLX40 against a cell line panel representing different forms of drug resistance. Cell survival was determined over 72 h using the FMCA assay in duplicate experiments. **(D)** Validation of VLX40 activity on 8226/Dox40 cells. Concentration-dependent effects of VLX40 on cell survival in RPMI 8226 (red line) and 8226/Dox40 (blue line) cell lines (triplicate samples). **(E)** Concentration-dependent effects of vincristine on cell survival in RPMI 8226 (red line) and 8226/Dox40 (blue line) cell lines. Survival in **(D**, **E)** was determined over 72 h using the FMCA assay. The results are expressed as percentage of the untreated control and presented as mean values +/- standard error of the mean (SEM) from three independent experiments.

We here used 8226/Dox40 myeloma cells as a model for drug resistance. Multiple mechanisms, including overexpression of P-glycoprotein, have been shown to contribute to the drug resistant phenotype [11-14]. A library of 3,000 chemically diverse compounds was used for screening of 8226/Dox40 and parental RPMI 8226 cells at a concentration of 1 μg/ml, and cytotoxic/antiproliferative activity was determined using FMCA (Figure 1A). One compound, RH02104 (Figure 1B) (subsequently denoted VLX40), demonstrated phenotype selective activity for the 8226/Dox40 subline.

A cell line panel of different origins, characterized by different mechanisms of drug resistance (Table 1), was tested for its sensitivity to VLX40 at 1 μg/ml. We found that VLX40 was not sensitive to multidrug resistance protein (MRP)- or topoisomerase II (Topo II)-mediated drug resistance (Figure 1C). Furthermore, the U-937/vcr cell line, associated with resistance to tubulin inhibitors, was almost as sensitive to VLX40 as parental U-937 cells (Figure 1C). Finally, immortalized human epithelial hTERT-RPE-1 cells were less sensitive to VLX40 at 1 μg/ml. Further hit confirmation in extended dose-response testing of VLX40 confirmed the relatively higher sensitivity of 8226/Dox40 compared to parental RPMI 8226 (Figure 1D), the difference in IC 50 being statistically significant (P < 0.05, Students *t*-test). In contrast, 8226/Dox40 cells are highly resistant to vincristine (Figure 1E). Based on these findings VLX40 was selected for further preclinical evaluation.

### VLX40 induces apoptosis in cancer cells

**Figure 2:**
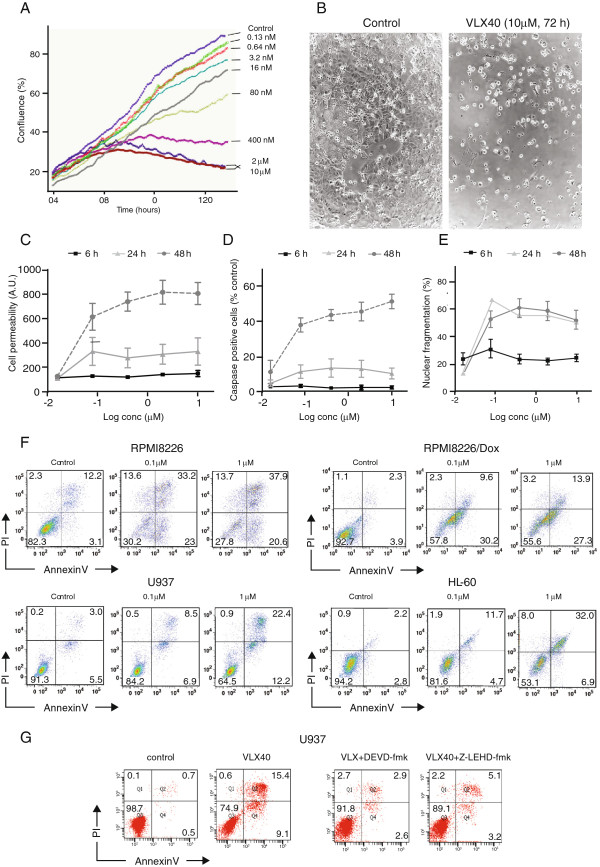
**VLX40 induces apoptosis in the MCF-7 breast cancer cell line.** In panel **(A)** cell growth kinetics were determined every hour during culture of MCF-7 tumor cells in 24-well plates. Cell confluence was determined by phase contrast time-lapse microscopy using an automated IncuCyte system. Representative phase contrast photomicrographs of control and VLX40 (10 μM) exposed cultures after 72 h are shown in panel **(B)**. Using Array Scan II the effects of VLX40 on membrane permeability **(C)**, DNA fragmentation **(D)** and caspase-3/7 activity **(E)** were evaluated and are shown over time (6-24 h). The results are expressed as percentage of the untreated control and presented as mean values + SEM from three independent experiments. Flow cytometry analysis of annexin V (x-axis) and propidium iodide (Y-axis) stained cells after 48 hrs exposure to VLX 40 in RPMI 8226 S, 8226/Dox40, U-937 and HL-60 cells **(F)**. In **(G)** the effect of VLX40 with and without caspase inhibitors DEVD-FMK and LEHD-FMK on annexin V staining is shown in U-937 cells.

We examined the response of both solid and hematological tumor cells to VLX40 (see further below). The response of the breast cancer cell line MCF-7 was studied using time-lapse phase contrast microscopy and multi-parameter analysis for cell death using Array Scan (Figure 2). A concentration-dependent effect on cell proliferation was observed (Figure 2A). Phase contrast images of treated cells showed a rounded-up morphology surrounded by a bright halo (Figure 2B). No increase in membrane permeability was observed at 6 h, whereas increases were observed at 24 and 48 h (Figure 2C). In parallel, we observed an increase in DNA fragmentation and caspase-3-like activity (using a DEVD-based substrate) at 24 and 48 h (Figure 2D and E).

Induction of apoptosis was confirmed by analysis of annexin V/propidium iodide staining (Figure 2F) in myeloma and myeloid leukemia cell lines (Figure 2F). RPMI 8226 and 8226/Dox40, U-937 and HL-60 cells were exposed to VLX40 for 24 hrs, stained and analysed by flow cytometry. Apoptosis was found to be reduced by inhibitors of caspase-3 and caspase-9, showing involvement of the intrinsic apoptosis pathway (Figure 2G).

### Identification of VLX40 as a tubulin active agent

**Figure 3:**
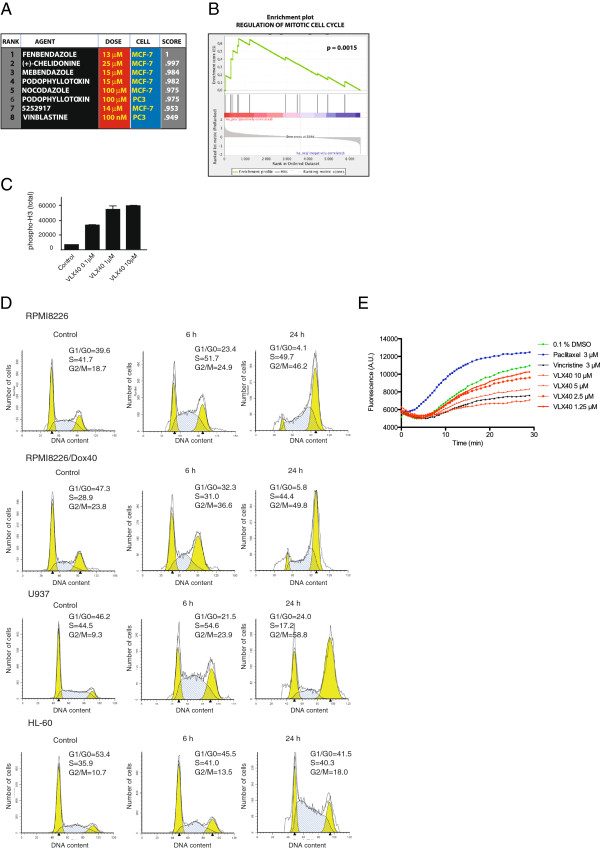
**VLX40 is a tubulin active agent. (A)** Microarray based mechanistic evaluation using Connectivity Map (cmap). MCF-7 cells were exposed to VLX40 for 6 h as described in experimental procedures. Out of the 6100 drug specific profiles in the data base, the eight most similar were all derived from compounds known to be tubulin inhibitors. 5252917 corresponds to N-(2-benzooxazol-2-yl-phenyl)-4-methyl-benzenesulfonamide. Score according to cmap data base. **(B)** Gene Set Enrichment Analysis (GSEA) shows significant up-regulation of genes involved in mitosis. The pre-ranked gene list (VLX40 exposed MCF-7 cells vs. untreated control) was compared to *a priori* defined and curated gene sets. The purpose of GSEA is to find out whether the *a priori* defined gene sets are significantly enriched towards the upper or lower end of the pre-ranked list. The p-value refers to the nominal p-value after 1000 permutations. **(C)** Phospho-histone H3 staining using Arrayscan VTI (total intensity) after exposure to VLX40 for 24 hrs in HCT 116 cells. **(D)** Analysis of cell cycle distribution after 24 hrs exposure to VLX40 in DAPI stained RPMI 8226, 8226/Dox40, U-937 and HL-60 cells. **(E)** Confirmation of tubulin inhibition as the mechanism of action of VLX40 using a cell free assay for tubulin polymerization. Vincristine (3 μM) and paclitaxel (3 μM) were used as reference compounds.

Mechanistic exploration was performed by measurement of gene expression of drug treated tumor cell cultures (Figure 3). The breast cancer cell line MCF-7 was exposed to 10 μM VLX40 or vehicle (DMSO) for 6 hours followed by microarray-based gene expression analysis. A drug specific query signature was generated and uploaded to the Connectivity Map (cmap), to find other compounds with similar mechanism of action. The VLX40 signature showed strongest similarity to known tubulin inhibitors such as fenbendazole, vinblastine, nocodazole and podophyllotoxin. In fact, all of the top seven compounds are tubulin inhibitors (Figure 3A) [31-34]. Gene set Enrichment analysis (GSEA) of genes induced by VLX40 showed significant association to mitosis (Figure 3B)

VLX40 induced a strong increase in phospho-histone H3 (Figure 3C) indicative of inhibition of mitosis and further cell cycle analysis demonstrated clear G2/M arrest in RPMI 8226 and 8226/Dox40 as well as in myeloid U-937 and HL-60 cells using flow cytometry (Figure 3D). The mechanistic hypothesis of VLX40 causing tubulin inhibition was subsequently confirmed by measuring tubulin polymerization *in vitro*. In this cell free assay both VLX40 and the reference compound vincristine (3 μM) clearly inhibited tubulin polymerization whereas paclitaxel (3 μM), as expected, increased polymerization activity (Figure 3E).

### Diagnosis-specific activity of VLX40 ex vivo

**Figure 4:**
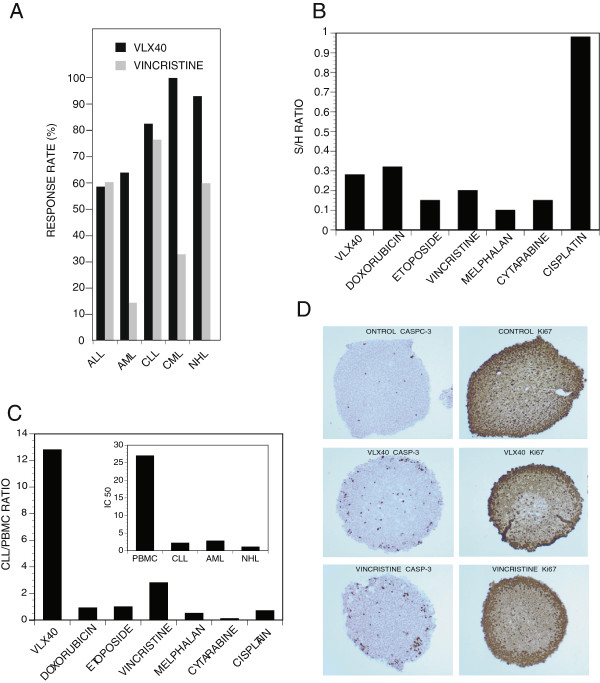
***Ex vivo *activity pattern of VLX40. (A)** The *ex vivo* response rate in a panel of primary cultures of patient tumor cells (PCPTC) representing a range of diagnoses (n = 98) is shown. The concentrations used were 3.4 μM of VLX40 and 1 μM of vincristine. See material and methods for details. **(B)** The solid tumor/hematological tumor activity ratio (S/H ratio) is displayed for VLX40 and six standard agents (n = 99). **(C)** The IC_50_ ratio between CLL (n = 9) and PBMC (n = 4) is shown for VLX40 and six standard drugs. **(D)** Caspase-3 induction in multicellular spheroids prepared from HCT116 colon carcinoma cells. Multicellular spheroids were treated for 24 h, fixed, sectioned and stained for active caspase-3. Note the induction of apoptosis preferentially at peripheral cell layers.

To examine the activity spectrum of VLX40, its cytotoxic effect was studied in 96 samples of primary cancer patient tumor cells (PCPTC) from patients with a variety of solid tumors and hematological malignancies as well as in four samples of primary lymphocytes from healthy donors (PBMC). Median IC_50_-values ranged from < 1 μM for diagnoses such as chronic lymphocytic leukemia (CLL), acute lymphocytic leukemia (ALL), acute myelocytic leukemia (AML), chronic myelocytic leukemia (CML) and lymphoma to > 34 μM for breast, ovarian, colon, lung and renal cancer samples (Table 2). PBMC displayed intermediate sensitivity to VLX40. The *in vitro* response rates to VLX40 at 3.4 μM for the PCPTC of various diagnoses is displayed in Figure 4A. Consistent with the IC_50_ patterns in cell lines, leukemic malignancies showed the highest response rates followed by ovarian carcinoma and breast cancer whereas colon and renal cancer demonstrated the lowest response rates. Vincristine was included as a reference compound demonstrating a similar activity spectrum with lymphocytic leukemias being most sensitive. However, myelocytic leukemias were clearly less sensitive to vincristine, contrasting the high in vitro response rate obtained with VLX40.

The relative effect of VLX40 and six standard cytotoxic drugs, in solid and hematological tumor samples, expressed as the solid/hematological (S/H) ratio is shown in Figure 4B. VLX40 had a ratio of 0.28 indicating a modest activity against solid tumors compared to cisplatin (S/H ratio 1.2). All the remaining drugs showed S/H ratios < 0.5. The results for the standard drugs are consistent with their main clinical use. To roughly estimate tumor cell specificity, drug effects were compared in cells from CLL and normal PBMCs. VLX40 demonstrated a significantly higher activity against the malignant phenotype with a PBMC/CLL median IC_50_ ratio of 12.2 (Figure 4C). Of the tested standard cytotoxic drugs only vincristine was more active in CLL than in PBMC.

To further evaluate and explain the relatively low activity of VLX40 on PCPTCs from solid tumors, which consists of multicellular clusters [21], we examined the ability of the compound to induce apoptosis of colon cancer cells grown as multicellular spheroids. As shown in Figure 4D, VLX40 showed a modest ability to induce apoptosis of cells in spheroids as evidenced by caspase-3 positive cells being mostly present in outer cell layers. The pattern was similar to that observed with vincristine (Figure 4D).

### VLX40 significantly inhibits in vivo growth of myeloid U-937 cells

**Figure 5:**
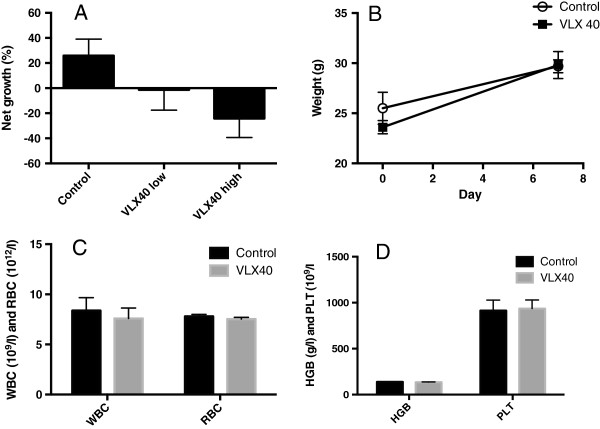
**VLX40 shows *in vivo *activity in hollow fiber cultures.** In **A**, the antitumoral effect of a single dose of VLX40 was investigated in mice (n = 8 per treatment group) carrying subcutaneous hollow fiber cultures of the myeloid cell line U-937. High and low refers to 2.0 and 0.5 μmol VLX40 per animal, respectively. The results are presented as net growth and expressed as mean value + S.E.M (n = 8). The difference between the high dose and control was statistically significant (p < 0.05, Student’s *t*-test). In panels **B**-**D** the effect of the high dose VLX40 on weight gain **(B)**, red and white blod cell count **(C)** and hemoglobin and platelet count **(D)** is shown.

*In vivo* activity of VLX40 was investigated in hollow fiber cultures of myeloid U-937 cells subcutaneously implanted in mice (Figure 5). After a single dose of VLX40 (2 μmol/animal) significant (p < 0.05) growth inhibition and tumor regression compared to vehicle treatment was observed. VLX40 showed no signs of toxicity at the doses tested.

## Discussion

Genomics-based target identification and screening using cell free systems has been the dominating principle in cancer drug discovery during the recent decade [6]. As an alternative to this approach the use of phenotype-cell-based screening may provide some distinct advantages [35]. We here performed a conditional screen with the aim of identifying compounds that are cytotoxic to multidrug resistant myeloma cells. A chemically diverse compound library was used for this purpose. The screening hit RH02104/VLX40 was the only compound that fulfilled the pre-determined criteria of a SI less than 50% in myeloma 8226/Dox40 and more than 50% in parental RPMI 8226 cells. In validation experiments VLX40 was found the difference was, albeit statistically significant, small. It can not be excluded that subtle differences in drug uptake and proliferation characteristics of the cell lines, not related to drug transporters, could contribute to the difference observed.

For exploration of mechanisms of action we used a bioinformatic approach using a drug specific gene expression signature to probe the cmap database [24]. The results indicated strong connections to tubulin-active agents. In vitro assays subsequently confirmed that VLX40 inhibits the polymerization of tubulin monomers and induces mitotic arrest.

A large number of tubulin active agents have been described in the literature, and some of these are important clinically used agents [36]. The majority of known tubulin inhibitors are natural products from many classes of organisms, suggesting that tubulin has been selected as a target by evolution at several independent occasions [36,37]. Interestingly, microtubule inhibitors have turned out to be significantly more successful in clinical practice compared to more recently developed mitosis-specific agents [38]. It has been suggested that the superior clinical efficacy of tubulin inhibitors is due to disruption of the function of microtubules in interphase cells [38].

Investigators have reported that microtubule inhibitors were identified in screens aimed to identify compounds directed at other targets, such as kinases [39], suggesting that tubulin polymerization may be a sensitive process that is easily targeted by a variety of chemical substances. Indeed, identification of tubulin inhibitors in screening diverse chemical libraries is not a rare event [26,40]. Nevertheless VLX40 showed a favorable pharmacological profile compared to vincristine being active against a multidrug resistant myeloma cell line with little sensitivity to other common forms of vinca alkaloid resistance.

VLX40 demonstrated a relatively narrow spectrum of activity in PCPTCs of various tumor types demonstrating activity preferentially in leukemias and lymphomas. Using PCPTCs with FMCA has demonstrated the ability to reflect tumor-type specific activity (25) as well as providing good clinical correlations (15,16). The spectrum of anti-leukemic activity was clearly distinct of that observed for vincristine; the largest difference being observed for AML cells which were sensitive to VLX40 but insensitive to vincristine. This spectrum of vinca alkaloid activity closely corresponds to clinical activity [30]. In contrast, VLX40 showed very limited activity on *ex vivo* solid tumor cells from breast, ovary, lung, colon and renal cancer patients. The reason for the low activity observed in the PCPTC solid tumor models may, at least partly, be due a to poor drug penetration in the latter model system, consisting of multicellular clusters [21]. This was supported by the modest antitumor activity obtained in the 3-D spheroid model cell line. However, in addition to poor penetration into the deeper cell layers also limited sensitivity and low proliferation of cells in these layers could contribute to the low solid tumor activity observed.

8226/Dox40 were originally selected for resistance to doxorubicin and show cross-resistance to mitoxantrone, acronycine, etoposide, and vincristine [11,15]. The resistant subline strongly overexpresses the MDR1 gene product P-gp170 [11,15]. A study using expression microarrays has confirmed MDR1 mRNA overexpression and also showed down-regulation of a number of apoptotic-regulators, including caspase-3, the proapoptic regulator BAD and TNF-receptors [12]. We have reported upregulation of STAT1-regulated genes in the 8226/Dox40 cell line [13,14]. While P-gp170 is clearly involved in vincristine resistance [15], the role of down-regulation of apoptotic regulators in the resistance of 8226/Dox40 to vincristine is more uncertain.

The high PBMC/CLL IC_50_ ratio indicates a potentially high therapeutic index *ex vivo*. It should be emphasized that both the PBMC/CLL ratio and S/H ratios are in vitro indicators for therapeutic index and clinical activity spectra and should be evaluated in relative rather than absolute terms. A ratio of 1 indicates equal sensitivity for PBMC vs. CLL and solid vs hematological activity, respectively. Thus, comparing and ranking different drugs with respect to these measures is a preferable way to utilize these indices. Indeed, the S/H index has previously been shown to correlate well to the clinical activity profile of standard cytotoxic cancer agents [41]. Both CLL and PBMC are largely non-proliferative under the present assay conditions. Furthermore, supporting these *ex vivo* findings VLX40 had significant *in vivo* activity against myeloid U-937 cells with no signs of toxicity. It should be noted that the hollow fiber is a very resistant *in vivo* tumor model requiring the drug to penetrate into fibers implanted deep subcutaneously, thus yielding a low false positive rate for cancer activity *in vivo* compared with other *in vivo* models [40]. However, the relatively low solubility of VLX40 in standard vehicles unfortunately limits the maximum dose that can be administered. Further work on improved formulations or analogue development may provide a potential future solution to this obstacle.

Chemically VLX40 is described as a 2-phenyl-4-hydroxyquinoline, which is a flavone-like element that has been used in medicinal chemistry previously, for example to design inhibitors of bacterial cell membrane pumps [42], or to inhibit cyclo-oxygenases [43]. Indeed several reports also demonstrate antiproliferative effects on human cancer cells, often as 2-phenyl-4-quinolones (isomers of 2-phenyl-4-hydroxyquinolines). For example, Hadjeri and co-workers synthesized a series of 5-hydroxy-2-phenyl-4-quinolones with potent antiproliferative activity in the NCI 60 cell line panel, and induced G2/M cell cycle arrest. Interestingly, the presence of a 5-hydroxy group (not present in VLX40) appeared to be important for these antiproliferative effects, which were not associated with microtubule inhibition [44]. However, others have shown that 2-phenyl-4-quinolones indeed do posses antimitotic activities, and that there is a good correlation between cytotoxicity of these compounds and their ability to inhibit tubulin polymerization [45-48].

The 2-phenyl-4-hydroxyquinolines are structurally unrelated to other tubulin inhibitors and may thus display other characteristics of importance for successful treatment, like spectrum of side effects or resistance. For example, vincristine is a substrate for both MRP and P-gp170, while VLX40 appears un-affected to both these mechanisms (Figure 1C). Furthermore, myeloblasts are often inherently resistant to vincristine, a feature that has been attributed to their high myeloperoxidase activity and generation of hydrogen peroxide by oxidation of hypochlorus acid subsequently leading to vinca alkaloid degradation [49]. The results of this study confirm the inherent resistance of AML cells to vincristine (Figure 4A), but not to the structurally different VLX40.

## Conclusions

In conclusion, the present study identified a novel tubulin active agent with retained activity in multidrug resistant models and which is active also against myeloid leukemia. VLX40 has a potential use as a cancer agent by virtue of its activity on drug resistant cells and may potentially be developed as an agent for AML. Further preclinical development will be required to evaluate its potential role as a novel prototype for future treatment of malignant diseases.

## Competing interests

JG, GW, PN and RL are co-founders and minor share-holders, MF and SL are minor share-holders, of Vivolux AB.

## Authors’ contributions

All authors were involved in designing experiments and interpreting data. MF, JG and and XW participated in most of the experiments and contributed equally to this work. RL, PN and SL have been involved in the overall design of the study and drafting major parts of the manuscript. MW and MJ performed the arrayscan experiments. LR designed and performed the primary screen. CA and MG created the screening database structure and provided bioinformatic support. SH performed the in vivo hollow fiber assay. GW contributed to the chemical aspects of the study with his expertise in medicinal chemistry. All authors read and approved the final manuscript.

## Pre-publication history

The pre-publication history for this paper can be accessed here:

http://www.biomedcentral.com/1471-2407/13/374/prepub
